# Metabolomics in Breast Cancer: From Biomarker Discovery to Personalized Medicine

**DOI:** 10.3390/metabo15070428

**Published:** 2025-06-23

**Authors:** Rosa Perestrelo, Catarina Luís

**Affiliations:** 1 CQM—Centro de Química da Madeira, Universidade da Madeira, Campus da Penteada, 9020-105 Funchal, Portugal; rmp@staff.uma.pt; 2 Faculdade de Ciências da Vida, Universidade da Madeira, Campus da Penteada, 9020-105 Funchal, Portugal

**Keywords:** metabolomics, breast cancer, biomarker discovery, personalized medicine

## Abstract

Breast cancer (BC) is a highly heterogeneous disease with distinct molecular subtypes, each exhibiting unique metabolic adaptations that drive tumor progression and therapy resistance. Metabolomics has emerged as a powerful tool for understanding cancer metabolism and identifying clinically relevant biomarkers guiding personalized therapeutic strategies. Advances in analytical techniques such as mass spectrometry (MS) and nuclear magnetic resonance (NMR) spectroscopy have enabled the identification of metabolic alterations associated with BC initiation, progression, and treatment response (dysregulated glycolysis, lipid metabolism, amino acid utilization, and redox homeostasis). This review aims to provide a comprehensive overview of the role of metabolomics in BC research, focusing on its applications in identifying metabolic biomarkers for early diagnosis, prognosis, and treatment response. It underscores how metabolomic profiling can unravel the metabolic adaptations of different BC subtypes, offering insights into tumor biology and mechanisms of therapy resistance. Ultimately, it highlights the promise of metabolomics in driving biomarker-guided diagnostics and the development of metabolically informed, personalized therapeutic strategies in the era of precision medicine.

## 1. Introduction

Cancer is a complex disease characterized by genetic, epigenetic, and metabolic alterations that drive tumor progression. The cells undergo profound metabolic reprogramming to support their rapid proliferation, survival, and resistance to therapy. According to International Agency for Research on Cancer (IARC) and global cancer statistics from 2022, it remains the major cause of death worldwide, with breast cancer (BC, 11.6% of the total cases) and lung cancer (LC, 12.4% of the total cases) being the most frequent cancers in women and men, respectively, in both cases and deaths [1,2]. Globally, it is estimated that by 2040, the burden from BC is predicted to increase to over 3 million new cases and 1 million deaths every year because of population growth [2]. BC is composed of multiple subtypes with distinct morphologies and clinical implications that lead to differences in response patterns to various treatment modalities and clinical outcomes [3].

To date, at least four molecular subtypes are commonly recognized, namely luminal A, luminal B, HER2-enriched, and basal-like (corresponding to triple-negative breast cancer, TNBC) [3]. The most prevalent subtype with the best prognosis is luminal A, which accounts for around 40% of cases, followed by luminal B at around 2–8% and TNBC at 15–20%, with worse clinical outcomes in terms of treatment [4].

In recent years, metabolomics has emerged as a crucial tool in cancer research, providing comprehensive insights into metabolic dysregulation associated with tumor development and progression. Metabolomics, the study of small-molecule metabolites in biological systems, has significantly enhanced our understanding of cancer metabolism, offering new avenues for biomarker discovery, disease stratification, and targeted therapies [5,6]. Moreover, the present review aims to discuss the significance of metabolomics in BC research, focusing on its contributions to understanding tumor metabolism, improving biomarker-driven diagnostics, and enabling metabolically tailored therapies, thus highlighting its pivotal role in personalized medicine.

## 2. Metabolomic Biomarkers in BC and Profiling Techniques

Metabolomics encompasses the study of small molecules, known as metabolites, within biological systems, providing crucial insights into cellular metabolism, helping researchers to understand disease mechanisms and discover biomarkers, leading to the development of targeted therapies [5]. In addition, it provides a snapshot of biochemical activity, reflecting genetic, transcriptomic, and proteomic influences on cellular function [7]. Moreover, metabolomic studies have become a key component of systems biology, offering essential information into disease mechanisms, biomarker discovery, and therapeutic strategies [8,9]. Various metabolomic profiling techniques enable comprehensive analysis of metabolic alterations associated with BC progression (Table 1).

**Figure 1:**
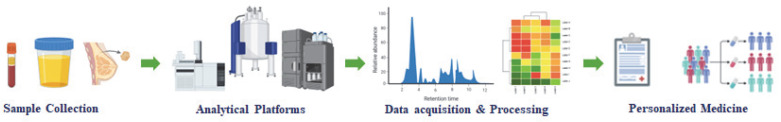
Integrative workflow of metabolomics in BC research.

As can be observed in Figure 1, these techniques comprise nuclear magnetic resonance (NMR) spectroscopy, mass spectrometry (MS)-based approaches, and chromatography-coupled methods. MS is a highly sensitive technique that allows the identification and quantification of metabolites, commonly coupled with separation techniques such as gas chromatography (GC) and liquid chromatography (LC), enhancing its ability to analyze complex biological samples [6].

MS-based approaches have been relevant in the identification of dysregulated metabolic pathways in BC, aiding in the discovery of potential therapeutic targets [19,20]. GC-MS is particularly effective in analyzing volatile and thermally stable metabolites, making it useful for profiling metabolic changes in biofluids and tissues [20]. As an example, Silva et al. [16] used cancer-free tissue to investigate the volatomic pattern and identify potential metabolites as biomarkers of the disease. The metabolites limonene, decanoic acid, acetic acid and furfural showed the highest sensitivity and specificity to discriminate BC patients. Another biofluid that offers a compelling, non-invasive alternative for BC biomarker discovery is saliva, and recent studies have begun to substantiate its diagnostic potential. Cavaco et al. [18] explored the potential of saliva as a sample for the investigation of BC biomarkers and found that 3-methyl-pentanoic acid, 4-methyl-pentanoic acid, phenol, and p-tert-butyl-phenol were able to discriminate between BC and CTL samples.

On the other hand, LC-MS provides a broader range of metabolite coverage, including lipids and polar compounds, offering a more comprehensive metabolic profile [21]. Anh et al. [13] used a multimodal omics approach, based on untargeted metabolomics and lipidomic analyses, to identify and validate potential biomarkers able to diagnose early-stage BC. In addition, it was verified that certain metabolites such as glutamate and glycochenodeoxycholate were altered in BC patients when compared with the control (CTL) group. HPLC, particularly when coupled with mass spectrometry, has been instrumental in profiling salivary metabolites, enabling the identification of distinct metabolic signatures associated with BC. For instance, a study utilizing capillary electrophoresis and LC-MS analyzed saliva samples from patients with invasive carcinoma and ductal carcinoma in situ, revealing significant differences in hydrophilic metabolite concentrations compared to healthy controls [17]. As another example, González Olmedo et al. [11] used plasma samples from patients with BC through untargeted liquid chromatography−high-resolution mass spectrometry (LC−HRMS) with the aim of identifying new candidate biomarkers that discriminated between the groups under study.

NMR spectroscopy has been used in metabolomics for BC research in recent years as a non-destructive analytical technique [22,23], allowing the identification and quantification of metabolites in biofluids, tissues, and cell extracts with high reproducibility [22]. In addition, proton (1H) NMR is particularly valuable due to its ability to detect a wide range of metabolites simultaneously. However, its sensitivity is lower when compared to MS-based techniques, limiting its detection capability for low-abundance metabolites [23,24]. These comprehensive approaches will represent a major step toward providing precision medical care, in which variability in genes, environment, and personal lifestyle is accounted for [25]. Many studies have been performed using these techniques or a combination of them with the aim of aiding in diagnostics or therapeutic interventions. A study performed by Wojtowicz et al. [26], for instance, used serum metabolites and metal ion profiling and identified distinct patterns differentiating BC patients from CTLs using unidimensional and bidimensional proton NMR experiments. Serum metabolomic analyses have shown promise in distinguishing between different BC subtypes. Studies have identified specific metabolite signatures in estrogen receptor (ER)-positive early BC patients, suggesting that post-operative serum metabolomic profiles could predict potential relapse [27]. Recent research has demonstrated that untargeted serum metabolomics, combined with machine learning tools, can concurrently detect multiple cancers, including BC, highlighting the potential of metabolomic profiling in multi-cancer screening applications [28,29]. In addition, early diagnosis is critical for improving prognosis and survival rates, but it remains challenging with traditional diagnostic tools. Metabolomic signatures can provide a non-invasive and highly sensitive approach for identifying BC at early stages. Several studies have identified specific metabolic alterations that could serve as early biomarkers for BC. For instance, a study by Yao et al. [30] highlighted that serum levels of choline metabolites, such as phosphocholine and glycerophosphocholine, were elevated in women with early-stage BC. These alterations reflect enhanced cell membrane turnover, a hallmark of cancer cell proliferation. Similarly, lactate, a byproduct of the Warburg effect, has been shown to be significantly elevated in early BC patients, potentially serving as an early metabolic marker [31]. Additionally, certain amino acids, such as glutamine and phenylalanine, showed altered concentrations in the serum of early-stage BC patients. These changes suggest that cancer cells may rely on specific metabolic pathways for survival and growth, even at early stages [32]. Despite the promising potential of metabolomic biomarkers, clinical validation remains a fundamental step before these biomarkers can be widely adopted in practice. Several reports have focused on validating metabolomic signatures through larger, more diverse patient cohorts to assess their accuracy, reproducibility, and utility in real-world clinical settings [33,34,35,36]. The validation is essential for ensuring that metabolomic biomarkers are not only statistically significant but also clinically relevant.

Regarding clinical applications, metabolomic profiling could be used in several areas, namely in diagnosis as an adjunct to imaging techniques like mammography or magnetic resonance imaging (MRI), helping to differentiate malignant from benign tumors or detect cancers skipped by conventional imaging methods [37]. In prognosis, it also could aid in providing additional information helping to predict disease recurrence or the likelihood of metastasis, thus guiding treatment decisions [38]. Regarding therapeutic monitoring, the changes in metabolites could serve as real-time biomarkers for monitoring treatment response. For example, a decrease in lactate levels may correlate with a favorable response to chemotherapy or radiation therapy [39,40].

Traditional BC diagnostic methods, including imaging (e.g., mammography, MRI), histology, and biopsies, have been essential for diagnosis and treatment planning but have limitations, particularly in early-stage detection, accuracy, and the invasiveness of procedures like biopsies. As for imaging techniques, mammography and MRI remain the gold standard for BC screening and diagnosis. Also, these methods are not precise, especially in dense BC, where mammography’s sensitivity decreases [41,42]. On the other hand, MRI has better sensitivity, but it is more expensive and less accessible. In contrast, metabolomic profiling could offer a less invasive, cost-effective, and complementary approach for early detection, potentially identifying metabolic changes before structural abnormalities appear on imaging scans. Histological examination of tissue samples from biopsies remains the standard method for BC diagnosis. Moreover, biopsies are invasive and carry risks of complications as they are not ideal for detecting early cancer or monitoring responses to therapy in real time. Metabolomics, on the other hand, could provide a non-invasive method for monitoring tumor metabolism and treatment responses through blood or urine samples [43].

One of the primary advantages of metabolomics over traditional methods is its ability to capture dynamic, real-time changes in metabolic pathways that may precede visible structural changes in the tissue. For example, metabolic alterations such as increased lactate or changes in amino acid profiles can be detected in the blood or urine before tumor growth becomes detectable through imaging techniques [38].

Another advantage is the ability to provide a more comprehensive, global analysis of tumor biology. While imaging and histology focus on specific aspects (e.g., tumor size or cellular morphology), metabolomics can give a broader picture of the biochemical environment of the tumor, potentially revealing insights into tumor aggressiveness, metastatic potential, and therapeutic targets [44]. Despite these advantages, metabolomics still faces challenges in terms of standardization, high-throughput analysis, and integration into clinical practice. The combination of metabolomics with traditional methods may offer the best of both worlds, enhancing diagnostic accuracy, improving early detection, and reducing the invasiveness of current procedures [8,20].

## 3. Metabolic Reprogramming and Therapy Resistance

Metabolites play a fundamental role in driving the growth and progression of BC through reprogramming cellular metabolism, allowing BC cells to meet the energetic and biosynthetic demands of rapid growth, survive in harsh tumor microenvironments, and metastasize to distant organs [45]. Understanding the complex interplay between metabolism and tumor biology can offer valuable insights into potential therapeutic strategies aimed at targeting metabolic vulnerabilities in BC. Beyond providing energy and biosynthetic precursors, metabolites act as signaling molecules that influence tumor progression (Table 2).

For example, the accumulation of certain metabolites, such as succinate, can activate pathways involved in angiogenesis and immune evasion [55]. Additionally, in BC, altered metabolic pathways can influence the tumor microenvironment, promoting immune suppression and resistance to therapies [56]. BC cells undergo several alterations in their metabolism, and one of the most well-known metabolic alterations in cancer is the shift toward aerobic glycolysis, also referred to as the Warburg effect [57,58]. Moreover, in normal cells, glucose is primarily metabolized via oxidative phosphorylation in mitochondria. However, in cancer cells, even in the presence of oxygen, glucose is converted to lactate through glycolysis. This shift allows cancer cells to generate ATP quickly and provides intermediates for biosynthetic pathways essential for tumor growth [59]. More specifically, in BC, this metabolic shift is critical for cell proliferation and survival. Increased glycolysis not only provides energy but also contributes to the accumulation of metabolic intermediates, such as ribose and nucleotides, which are necessary for DNA and RNA synthesis [58]. Furthermore, lactate produced by glycolysis may be exported into the tumor microenvironment, contributing to an acidic environment that promotes tumor invasion and metastasis [40]. Additionally, fatty acid metabolism plays a crucial role in BC progression where tumor cells often exhibit increased fatty acid synthesis to support membrane biogenesis and energy storage. In addition, the oxidation of fatty acids in mitochondria provides energy and supports the survival of cancer cells, especially in hypoxic regions of the tumor [40]. Fatty acid synthase (FASN), a key enzyme in fatty acid biosynthesis, is often overexpressed in BC and is associated with poor prognosis [58]. In BC, FASN expression has been linked to increased tumor growth, metastasis, and resistance to chemotherapy [60,61]. Targeting fatty acid metabolism through the inhibition of FASN has shown promise in preclinical models and is being explored as a potential therapeutic strategy [62]. In addition to glucose and lipids, amino acids are essential for the growth and survival of BC cells. The amino acid glutamine is of particular importance, as it serves as a critical source of nitrogen for biosynthesis and as a fuel for the tricarboxylic acid (TCA) cycle. Glutamine metabolism is often upregulated in cancer cells to support anaplerotic reactions that replenish TCA cycle intermediates, which are vital for energy production and biosynthesis [63]. BC cells also rely on the conversion of the amino acid serine into glycine, which is necessary for the synthesis of nucleotides, lipids, and proteins required for cell division [64]. Alterations in amino acid metabolism provide not only an energy source but also the building blocks needed for rapid tumor growth. The tumor microenvironment is often characterized by low oxygen levels (hypoxia), which further drives metabolic reprogramming. In BC, hypoxia leads to the activation of hypoxia-inducible factors (HIFs), which upregulate genes involved in glycolysis, angiogenesis, and cell survival [65]. HIF-1α, a key regulator of the hypoxic response, induces the expression of genes involved in glucose uptake and glycolysis, promoting a shift toward anaerobic metabolism that supports tumor growth in oxygen-deprived regions. Moreover, the hypoxic tumor microenvironment induces metabolic adaptations that allow tumor cells to thrive despite nutrient shortages. Hypoxia enhances fatty acid oxidation and the use of alternative metabolites, which helps to maintain cellular energy levels and supports survival during metastasis [45].

In BC, this metabolic shift supports the rapid energy and biosynthesis requirements of rapidly proliferating cells. In addition, glycolysis produces not only ATP but also important metabolic intermediates that are essential for macromolecule synthesis, such as nucleotides, amino acids, and lipids, thereby facilitating cell division and tumor growth [66,67]. The increased glycolytic flux observed in BC is often accompanied by an upregulation of key enzymes such as hexokinase II (HKII), pyruvate kinase M2 (PKM2), and lactate dehydrogenase A (LDHA). These enzymes are involved in the regulation of glucose uptake, glycolysis, and lactate production, respectively [68]. Elevated lactate production, in turn, can create an acidic microenvironment that promotes tumor invasion and metastasis [40]. Moreover, the Warburg effect is closely linked with key signaling pathways such as the PI3K-AKT-mTOR pathway, which not only supports metabolic reprogramming but also drives tumor progression [67]. As already mentioned, BC metastasis is a complex process that involves the dissemination of tumor cells to distant organs, where they adapt to the new microenvironment. Metabolic alterations are thought to play a significant role in metastasis by allowing tumor cells to survive in unfavorable conditions. One prominent example is the role of fatty acid metabolism in metastasis. Elevated fatty acid oxidation (FAO) has been linked with enhanced metastatic potential in BC, where FAO supports energy production and reduces oxidative stress, promoting cell survival in distant tissues [69,70]. Furthermore, abnormalities in mitochondrial function and metabolic flexibility enable BC cells to adapt to nutrient-poor environments, a key characteristic of metastatic spread [71].

The ability of BC cells to alter their metabolism in response to different microenvironments is also a hallmark of metastasis. For instance, the epithelial-to-mesenchymal transition, a process that allows cancer cells to gain migratory and invasive properties, has been shown to correlate with metabolic shifts, including enhanced glycolysis and lipid biosynthesis [72]. These metabolic changes support the energy requirements for migration and invasion, which are essential steps in metastasis.

The altered metabolic state of BC cells also contributes to the development of resistance to chemotherapeutic agents and targeted therapies. Metabolic reprogramming enables tumor cells to adapt to therapeutic stress, and certain metabolic pathways are directly involved in mediating drug resistance (Table 3).

For example, glycolytic intermediates can influence the activity of ATP-binding cassette (ABC) transporters, which are involved in the efflux of chemotherapeutic agents from the cell, thus reducing drug efficacy [76,77].

Additionally, the accumulation of reactive oxygen species (ROS) resulting from mitochondrial dysfunction and altered metabolism can modulate signaling pathways associated with cell survival and resistance. For instance, the stabilization of hypoxia-inducible factor 1-alpha (HIF-1α) under metabolic stress has been shown to promote resistance to both chemotherapy and targeted therapies by activating pro-survival pathways [77,78,79]. In BC, increased glycolytic activity, coupled with enhanced lactate production, has been linked to resistance to trastuzumab in HER2-positive BC, emphasizing the importance of targeting metabolic pathways in combination with traditional therapies to overcome resistance [80].

Regarding treatments, chemotherapy and targeted therapies often rely on disrupting cancer cell proliferation and survival mechanisms, but tumor cells can adapt by reprogramming their metabolism to withstand drug-induced stress. One key metabolic alteration associated with resistance to chemotherapy is the shift toward glycolysis, even in the presence of oxygen, a hallmark of the Warburg effect. The increased glycolytic activity not only provides tumor cells with an immediate source of energy but also generates metabolic intermediates that support cellular repair and biosynthesis, thus aiding in the recovery from drug-induced damage [78]. In addition to glycolysis, the rewiring of lipid metabolism has been implicated in chemotherapy resistance. Fatty acid oxidation (FAO), for example, is upregulated in several cancers, including BC, and has been shown to contribute to chemotherapy resistance by promoting mitochondrial function and energy production, thereby supporting cell survival during drug treatment [69,81,82]. This metabolic shift helps BC cells evade apoptosis induced by chemotherapeutic agents such as doxorubicin, which typically target rapidly dividing cells [83,84]. Several mechanisms of resistance to chemotherapy and targeted therapies are driven by metabolic changes that alter drug response. One of the most prominent mechanisms mentioned before is the enhanced activity of ATP-binding cassette (ABC) transporters, which are responsible for pumping chemotherapeutic drugs out of cells. The increased glycolytic flux associated with the Warburg effect leads to elevated levels of intracellular ATP, which, in turn, upregulates the expression of these transporters, resulting in decreased drug accumulation and diminished efficacy [70]. Furthermore, metabolic adaptations in response to hypoxia, a common feature of the tumor microenvironment, contribute to resistance. HIFs, which are stabilized under low-oxygen conditions, promote metabolic shifts toward glycolysis and FAO. HIF-1α, in particular, not only drives glycolytic flux but also enhances cell survival by regulating the expression of pro-survival genes such as Bcl-2 and by increasing the synthesis of anti-apoptotic molecules [85]. This hypoxia-driven metabolic reprogramming has been linked to resistance against a wide range of chemotherapeutic agents and targeted therapies, including HER2 inhibitors and hormonal therapies in BC [86,87].

In addition to altering glycolytic and lipid metabolism, BC cells can also adapt their mitochondrial metabolism to resist oxidative stress induced by chemotherapy. Mitochondrial dysfunction in response to treatment can lead to decreased ROS production, which otherwise might trigger cell death. On the other hand, altered mitochondrial dynamics, such as the increased fusion of mitochondria, have been observed in resistant BC cells and contribute to their survival [86,88].

Given the central role of metabolic reprogramming in therapy resistance, targeting metabolic pathways represents a promising strategy to overcome resistance in BC. Several potential metabolic targets have emerged, including enzymes involved in glycolysis, lipid metabolism, and mitochondrial function. Regarding glycolytic enzymes, inhibiting key enzymes in the glycolytic pathway, such as hexokinase 2 (HK2) or pyruvate kinase M2 (PKM2), can reduce the glycolytic flux and limit the availability of metabolic intermediates necessary for tumor survival. Studies have shown that targeting HK2 with small-molecule inhibitors sensitizes BC cells to chemotherapy and improves treatment outcomes [75,89]. Additionally, inhibitors of PKM2 have been shown to reduce tumor growth and enhance the effectiveness of chemotherapies [90].

In fatty acid metabolism, targeting FAO is another promising approach to overcoming drug resistance. The use of FAO inhibitors, such as etomoxir, has demonstrated the potential to sensitize BC cells to chemotherapeutic agents, particularly by impairing mitochondrial energy production and promoting cell death [91]. Furthermore, combining FAO inhibitors with chemotherapy has been shown to inhibit metastasis in preclinical BC models, highlighting the importance of lipid metabolism in both primary tumor growth and metastasis. Another target pathway could be mitochondrial metabolism, which plays a key role in drug resistance, particularly in response to oxidative stress. Mitochondrial uncouplers, which disrupt the mitochondrial membrane potential and reduce ROS production, are being explored as potential agents to enhance the efficacy of chemotherapy. In preclinical models, mitochondrial uncouplers have been shown to improve the sensitivity of resistant BC cells to drugs like paclitaxel and docetaxel [92,93]. Additionally, targeting mitochondrial dynamics through inhibition of fission or promoting mitochondrial apoptosis has also been proposed as a strategy to overcome resistance [84]. In addition, HIF-1α plays a central role in regulating metabolic adaptations in response to hypoxia; targeting HIF-1α or its downstream signaling pathways may offer a way to overcome resistance. Inhibitors of HIF-1α have shown promise in preclinical models, particularly in combination with chemotherapy, by reducing glycolytic flux and enhancing the sensitivity of BC cells to treatment [94,95].

## 4. Metabolite-Based Therapeutic Strategies

BC cells exhibit metabolic reprogramming to support unlimited growth, evade immune recognition, and develop therapy resistance. The adaptation is marked by altered energy production, biosynthesis, and redox balance, exploitable targets for targeted therapy. A few of the most critical metabolic processes and therapeutic approaches are glycolysis (Warburg effect), oxidative phosphorylation, glutaminolysis, and lipid metabolism [96,97]. Even in oxygen-rich settings, BC cells, especially TNBC, display the Warburg effect, which is a preference for aerobic glycolysis over oxidative phosphorylation [98]. Hypoxia and oncogenic signaling (e.g., hypoxia-inducible factor 1-alpha (HIF-1α), c-Myc) induce this metabolic change, which facilitates quick ATP generation, biosynthetic precursor synthesis, and adaptability to nutrient-poor microenvironments [57]. In TNBC, where the Warburg effect is closely linked to the development of the illness, emerging therapies focus on using metabolic vulnerabilities and interfering with oncogenic drivers (such as HIF-1α and c-Myc) [57,94,99,100]. These oncogenic factors reprogram cellular metabolism to promote tumor growth and survival by enhancing glycolysis and other metabolic pathways. Preliminary data support the prognostic utility of these biomarkers (41.4% for HIF-1α and 55.2% for c-Myc) for TNBC, being significantly associated with clinical parameters, including tumor size, histological grade, lymph node status, and TNM stage [101]. Nevertheless, external validation in larger cohorts and integration with existing clinical models (e.g., pN stage, imaging features) are critical [102].

Another intriguing treatment approach for cancer is to target glutamine metabolism, specifically by inhibiting glutaminase (GLS1). The metabolic flexibility of cancer cells, particularly those in TNBC, allows them to use glutamine as an alternate energy source in response to nutritional stress. GLS1 contributes to cellular bioenergetics and biosynthesis by converting glutamine to glutamate, which then enters the tricarboxylic acid cycle (TCA cycle). Cancer cells undergo decreased glutamine metabolism when GLS1 is inhibited, which results in a decrease in cell division and an increase in apoptosis susceptibility [98,103]. Clinical research into CB-839 (Telaglenastat), a powerful GLS1 inhibitor, as a targeted treatment for glutamine-dependent malignancies is supported by the fact that it has strong anticancer effects in xenograft models and significant antiproliferative activity in TNBC via lowering glutamine metabolism [103]. To further improve efficacy and overcome resistance mechanisms in BC, especially in aggressive subtypes of TNBC [104], GLS1 inhibitors (e.g., CB-839, BPTES) are also being actively investigated in combination with other therapies, including immune checkpoint inhibitors, chemotherapy [105], targeted therapies [98,106] and metabolic modulators [98]. These combinations target resistance pathways while taking advantage of the metabolic weaknesses of cancer cells.

As vital building elements for membrane synthesis, energy production, and cell communication, lipid metabolism is important in BC, especially in hormone receptor-positive (HR+) subtypes. A promising treatment approach is to interfere with lipid metabolism, especially using fatty acid synthase (FASN) inhibitors or interfering with cholesterol metabolism [107,108,109]. In preclinical studies, Fasnall, a selective thiophenopyrimidine-based inhibitor of fatty acid synthase (FASN), has shown a strong antitumor effect, especially in HER2-positive BC. Fasnall dramatically decreased tumor volume in mouse models when paired with carboplatin, suggesting the possibility of combination treatments in a human scenario [110]. In another research, the effectiveness of combining TVB-2640 with trastuzumab and paclitaxel in patients with HER2-positive BC is being evaluated in a noteworthy Phase II experiment. As a treatment option for TNBC brain metastases, this combination therapy merits more research because it has the potential to decrease tumor development [111].

On the other hand, dietary interventions can modulate cancer metabolism, potentially enhancing standard treatments and improving patient outcomes. In this sense, the ketogenic diet (KD), a high-fat, low-carbohydrate diet with suitable protein and calories, is a bright prospect to target these metabolic alterations in tumor cells. According to recent studies, the KD may prevent the growth of tumors, shield healthy cells from radiation or chemotherapy, increase the toxicity of chemotherapy to cancer cells, and reduce inflammation [51,112,113]. Buga et al. [51] studied the feasibility, safety, and metabolic benefits of a KD in metastatic BC patients undergoing chemotherapy. The results obtained highlight the potential therapeutic benefits in enhancing body composition, glucose metabolism, and insulin resistance, all of which may improve the prognosis of BC. Schmidt et al. [48] verified that in TNBC models, lowering systemic glucose via pharmacologic (metformin) and dietary (KD) means dramatically reduced tumor development and extended longevity. Moreover, dietary fatty acids (e.g., eicosapentaenoic acid, docosahexaenoic acid) may be vital in preventing BC and even enlightening the quality of life for survivors when anticancer medications, which are specifically developed to kill cancer cells and reduce the tumor burden, fail to work against certain stages of tumorigenesis, according to epidemiological studies conducted over the past few decades [47,48,54,114,115,116]. A daily increase of 0.1 g of omega-3 fatty acids has been demonstrated to reduce the risk of BC by 5%, and a diet high in omega-3 fatty acids can increase patient survival for those who already have the disease [52]. Luo et al. [47] highlighted omega-3 fatty acids as a promising dietary and therapeutic approach to target cancer stem cell metabolism in BC, since eicosapentaenoic acid and docosahexaenoic acid (omega-3 fatty acids) effectively downregulated lipogenic enzyme expression. Another dietary intervention is intermittent fasting, which consists of long periods (e.g., 16–48 h) of slight to no caloric consumption, constantly irregular with periods of ad libitum intake [117], which is a viable approach to improve the efficacy and acceptability of chemotherapy. In nutrient deprivation, healthy cells are protected from chemotherapy, which instructs them to control their growth and division. On the other hand, cancer cells’ mutations reduce their ability to adapt to the unique circumstances that arise during fasting, making tumor cells more vulnerable to chemotherapy during the fasting cycle [53,118]. Son et al. [49] verified that intermittent fasting lowered cyclin B1 and vimentin levels, indicating suppression of TNBC cell proliferation and metastasis, particularly in obese patients.

These findings highlight the critical role that metabolic interventions (e.g., dietary changes, targeted therapies) play in slowing the spread of BC, especially in aggressive subtypes like TNBC and HER2-positive BC. Through the use of FASN and GLS1 inhibitors, ketogenic diets, omega-3 fatty acids, and intermittent fasting, researchers and clinicians are taking advantage of metabolic vulnerabilities to discover new, supplemental therapeutic approaches that could improve treatment efficacy, reduce resistance, and improve patient outcomes. This opens the door for integrated metabolic-targeted approaches in the management of BC.

## 5. Metabolomics for Personalized Medicine

Metabolomics, the comprehensive characterization of metabolites in biological systems, has emerged as a pivotal omics approach to further personalized therapeutic strategies in BC by identifying potential biomarkers and metabolic alterations, predicting response to treatment, and monitoring treatment response [9,119,120]. Understanding disease processes, finding therapeutic targets, and clarifying the mode of action of drugs are just a few of the fundamental drug research applications where metabolomics, with its sophisticated analytical tools (e.g., NMR, MS), offers unparalleled benefit. Moreover, metabolomics predicts pharmacokinetics, pharmacodynamics, and medication response, which significantly speeds up the drug development process [121]. Metabolomics is a relatively new and rapidly growing field of study that supports proteomics and genomics and is becoming increasingly significant in studies seeking to uncover biomarkers and improve personalized therapy [122,123], as shown in Table 4.

Understanding the metabolic variations among molecular BC subtypes (LA, LB, HER2+, TN) may help develop new subtype-specific treatment approaches, as the term refers to a group of distinct diseases. This is particularly true if metabolite alterations are assessed within the larger framework of the network of enzymatic reactions and pathways [119,120,125]. Díaz-Beltrán et al. [125] used an untargeted LC-HRMS metabolomics strategy to distinguish BC subtypes, and identified biomarker panels containing five candidates for LA, seven for LB, five for HER2+, and three for TN. These biomarkers demonstrated strong diagnostic potential, with an area under the receiver-operating characteristic (AUROC) curve exceeding 0.85, contributing to precision medicine by improving BC diagnosis and enabling subtype-specific targeted therapies. Unfortunately, no one therapy can be used to treat every patient; as a result, the clinic must predict the therapeutic response while matching the right patients with the right treatment. To find promising and useful biomarkers to direct patient care and match the right treatment, further methods are required. Metabolite profiling is a gratifying and economical method that allows for a comprehensive and systematic analysis of metabolites. It may also be used to predict and monitor medication response, find therapeutic targets for drug discovery, and provide tailored care to lessen the burden of disease. A patient’s pharmacological phenotype is closely correlated to the application of small metabolites to predict specific reactions to medication therapy. When interpreting the metabolome data, it can provide more information than other omics data. Additionally, it makes it possible to investigate promising models for forecasting therapy response. By forecasting pharmacological reactions to specialized therapy, small-molecule metabolites can be used for patient diagnosis and prognosis. Furthermore, metabolic fingerprints can yield a wealth of information from targeted medication therapy or metabolic pathways [9,126,133,134,135]. Díaz et al. [127] identified potential metabolic biomarkers predictive of neoadjuvant chemotherapy response in TNBC, since docosahexaenoic acid and secondary bile acids at elevated levels were related to enhanced response. On the other hand, glycohyocholic and glycodeoxycholic acids efficiently distinguished patients in terms of treatment response and survival. For LB and HER2+ subtypes, differences relating to the patient, as well as duration, were noticed, in which tryptophan decreased and LysoPE (22:6) increased in the case of HER2+ but without an isolated association with therapy response. Irajizad et al. [135] used deep learning to build a predictive model using polyamines and nine other metabolites, which would ideally be capable of classifying TNBC patients with low chances of responding to neoadjuvant chemotherapy and guiding alternative treatment modalities.

A multifaceted understanding of disease biology is produced by combining metabolomics with other omics techniques like transcriptomics, proteomics, and genomics. This is especially useful in precision medicine since it significantly advances our knowledge of the pathophysiology of the target clinical phenotype and the disease mechanisms [136,137,138,139]. Numerous studies have been conducted to enhance the advantages of customized healthcare by merging omics approaches. For instance, combining transcriptomic and metabolomic data identifies metabolic dependencies brought on by certain genetic changes, offering important new information on BC metabolism and possible treatment options [46,140]. Bonanomi et al. [141] integrated transcriptomics and metabolomics to explore the role of C-terminal binding protein 2 (CtBP2) as an NADH sensor, linking metabolism to epigenetic transcriptional regulation in TNBC cells. The results validate CtBP2 as a key modulator of metabolic–epigenetic crosstalk, suggesting that its inhibition can disrupt cancer cell proliferation through redox balance and metabolic regulation; thus, CtBP2 could be a potential therapeutic target in TNBC. On the other hand, Zhang et al. [74] investigated biomarkers of neoadjuvant activity of a TCbHP regimen (taxane, carboplatin, trastuzumab, and pertuzumab) in HER2+ BC patients with metabolomics and transcriptomics. The data provided deeper mechanisms of resistance to trastuzumab and could potentially be beneficial in the design of new therapeutic targets in unsatisfactory-treatment-response patients. Xiao et al. [142] performed a comprehensive study that profiled the polar metabolome and lipidome in 330 TNBC samples and 149 paired normal breast tissues, creating a large metabolomic atlas of TNBC. By integrating metabolomic, transcriptomic, and genomic data, TNBCs were classified into three metabolomic subgroups, C1 (enriched in ceramides and fatty acids), C2 (elevated oxidation and glycosyl transfer metabolites), and C3 (least metabolic dysregulation), providing potential targets for personalized therapeutic interventions. On the other hand, the integration of metabolomics and proteomics in precision medicine enhances the understanding of tumor biology, leading to better biomarker discovery, treatment response prediction, and therapeutic advancements. An et al. [12] integrated metabolomics, proteomics, and machine learning to identify specific metabolic signatures in human plasma for BC diagnosis. A panel of 47 plasma metabolites, including sphingomyelins, glutamate, and cysteine, showed potential as diagnostic biomarkers for BC. Sharaf et al. [123] evaluated the effects of tamoxifen, trastuzumab, and a combination of both on the proteomic and metabolic signature of TNBC (BT-474) cells to assess potential overtreatment concerns. The anticancer drugs significantly impacted key signaling pathways in triple-positive BT-474 cells, regulating cell growth, apoptosis, proliferation, and chemoresistance. The data suggests that treatment intensity should be reevaluated to avoid overtreatment for this patient group.

Ongoing research and clinical trials continue to enhance metabolomics-based interventions, bringing precision medicine closer to routine clinical practice for BC patients [129,130]. Blood-derived metabolite biomarkers have been found using metabolomic analysis to forecast the efficacy of neoadjuvant treatment in TNBC. He et al. [129], using NMR-based metabolomics analysis, recognized three noteworthy metabolic pathways that might distinguish patients with partial, complete, or stable responses to neoadjuvant chemotherapy in TNBC. According to the results, metabolic phenotyping may be used to assess the appropriateness of neoadjuvant chemotherapy, enabling tailored treatment plans for TNBC patients and so minimizing needless treatment exposure. After patients had six or eight cycles of anthracycline–docetaxel, another investigation utilizing LC-MS found nine metabolites that could accurately distinguish between partial and stable responses to neoadjuvant chemotherapy in TNBC, with high sensitivity (100%) and specificity (81.2%) [130]. Moreover, the effectiveness of therapy is impacted by variations in tamoxifen metabolism across individuals, as revealed by metabolic investigations. In comparison to sensitive cells, resistant cells had higher amounts of glutamine, taurine, glutathione, and xanthine, according to research on tamoxifen-resistant MCF-7 BC cell lines [73]. According to these results, metabolomics may be able to assist in modifying dosage or locating substitute therapies for non-responders.

## 6. Challenges and Future Directions in Metabolomics for BC

Metabolomics research in BC encounters enormous technical and analytical hurdles that must be surmounted to unlock its clinical promise. One of the biggest challenges is the complexity and heterogeneity of biological samples [143]. Metabolite levels are highly dynamic and influenced by numerous factors such as diet, lifestyle, medicines, gut microbiota, circadian rhythms, and physiological states that contribute to the variability of the metabolite levels, hence making standardization difficult [119]. Tumor heterogeneity complicates the identification of reliable metabolic biomarkers further because BC subtypes, e.g., ER-positive and triple-negative, possess distinct metabolic signatures [119]. Heterogeneity also exists among patients and even within the same tumor, so it is difficult to find shared metabolic markers. Moreover, sample handling, storage conditions, and collection significantly influence the stability of metabolites, so strict protocols for minimizing degradation and ensuring reproducibility are needed [119]. Another major bottleneck in metabolomics research is data integration and interpretation, particularly when metabolomics is being combined with other omics approaches. The high-dimensional data generated are difficult to interpret and need sophisticated computational tools and statistical methods to extract meaningful information. Establishing correlations between metabolic changes and genetic or proteomic alterations is complex, requiring robust machine learning algorithms and AI-driven analytics [144,145,146]. A real-world study showed that AI-supported screening achieved a BC detection rate of 6.7 per 1000, which was 17.6% higher than the 5.7 per 1000 rate in the control group, demonstrating statistical superiority. The recall rate in the AI set was 37.4 per 1000, slightly lower than the 38.3 per 1000 in the control set, demonstrating non-inferiority. AI also enhanced screening effectiveness, with a greater positive predictive value of recall (17.9% vs. 14.9%) and biopsy (64.5% vs. 59.2%) [147]. Nevertheless, these models frequently encounter constraints in interpretability and generalizability across several cohorts. In addition, identification of biologically relevant metabolites from noise and interfering factors remains a fundamental challenge needing rigorous validation by independent experiments. Alternatively, instrumentation and sensitivity also pose inherent limitations in metabolomics research. MS and NMR are the most important analytical instruments, but challenges remain with the detection of low-abundance metabolites. While MS is very sensitive, it is plagued by ion suppression, which degrades accuracy and reproducibility [146]. On the other hand, NMR provides quantitative information but with less sensitivity, limiting the detection of low-abundance metabolites [148]. Metabolite identification remains a bottleneck since spectral reference databases are not comprehensive, hindering the characterization of unknown or new metabolites. Batch effects and inconsistencies in calibration of the instruments also introduce variability, which emphasizes the need for standardized research methodologies in research laboratories. MS and NMR integration are highly encouraged together with MS sensitivity and NMR structural specificity to ensure clinical needs are met [149]. On this point, AI-driven workflows can now perform cross-validation of biomarkers from both approaches with greater confidence. Metabolic transformation is closely tied to enzyme activity, and this relationship holds significant potential for identifying novel diagnostic biomarkers and therapeutic targets in BC. Metabolomic abnormalities often reflect underlying alterations in metabolic enzyme expression or activity. For instance, upregulation of choline kinase (CHKA) can contribute to phosphocholine accumulation, a metabolic hallmark of aggressive breast tumors [150]; similarly, mutations or overexpressed isocitrate dehydrogenase (IDH) can produce oncometabolites that promote tumor progression [151].

Much of the existing metabolomics studies are based on small cohorts of patients, limiting their reproducibility, generalizability, and clinical importance. To address these problems, extensive, multicenter research is needed to support metabolomics-based biomarkers in diverse populations and therapy protocols to determine their clinical significance, reproducibility, and reliability by harmonizing sample collection, processing, and analysis methods, investigating metabolic distinctions between different BC subtypes and response to treatment, correlating clinical trial data with the results to assess their predictive value for guiding personalized treatments, developing non-invasive diagnostic techniques such as liquid biopsies, monitoring disease development and recurrence, and eventually extending the potential of precision medicine to improve early detection, therapeutic decision-making, and patient outcomes in BC management. A multicenter study of 1947 participants recognized a five-metabolite diagnostic panel (glutamate, erythronate, docosahexaenoate, propionylcarnitine, and age) that outperformed traditional markers like CA15-3, proving robustness across ethnicities and geographic areas with an AUC of 0.954 in training cohorts and 0.834–0.954 in validation cohorts, guaranteeing early detection significance by including 75.8% early-stage (TNM I/II) samples, confirming BC specificity by testing against seven other cancer types, and highlighting the benefit of large cohorts in mitigating biases linked with small studies with nonexistent statistical power to explain lifestyle, genetic, and environmental variables [152]. In another study, large cohorts enabled granular analysis of BC heterogeneity, including the classification of TNBC into three metabolically distinct clusters: C1 (ceramide/fatty acid-enriched), linked to luminal androgen receptor (LAR) tumors and targetable via sphingosine-1-phosphate (S1P) inhibition; C2 (oxidative/glycosyl transfer), associated with high-risk BLIS tumors with elevated N-acetyl-aspartyl-glutamate; and C3 (low dysregulation), which has a better prognosis and is distinguishable via machine learning [152,153].

Metabolomics is rapidly advancing, offering new possibilities for improving BC detection, prognosis, and treatment. Emerging technologies and computational tools are enhancing metabolomics-based approaches’ accuracy, sensitivity, and clinical relevance. Key developments in this field include high-resolution metabolomics, namely high-resolution magic angle spinning (HR-MAS); magnetic resonance spectroscopy (MRS), which can identify elevated levels of metabolites like choline and glycine, which correlate with tumor aggressiveness and imaging parameters [154]; AI-driven data analysis; non-invasive diagnostics; and precision medicine applications. The development of AI-based computational methods will improve metabolite identification and biomarker discovery by enabling more efficient data analysis and interpretation. Expanding and refining standardized reference databases will facilitate more accurate metabolite characterization and cross-study comparability. Furthermore, bridging the gap between research and clinical application requires large-scale validation studies, regulatory frameworks, and real-world data integration. By overcoming these obstacles, metabolomics research has the potential to transform BC diagnostics and treatment, paving the way for more personalized and effective therapeutic strategies [155]. While plasma and cell line metabolomic profiles are well documented in BC, the metabolomic landscape of small extracellular vesicles (sEVs), including exosomes, is still an emerging area of investigation. These vesicles carry a subset of metabolic components that reflect the metabolic state of their parent cells and may contribute to tumor progression and communication with the microenvironment [156]. A few preliminary studies have begun to explore sEV metabolomics in breast cancer models—for example, distinct diacylglycerol enrichment in sEVs from triple-negative breast cancer lines and elevated 27-hydroxycholesterol in sEVs from estrogen-receptor-positive cells [156]—while untargeted profiling has uncovered specific metabolites such as lysoPC 22:6 and N-acetyl-L-phenylalanine in sEVs from MDA-MB-231 versus non-tumorigenic cells [157]. Moreover, clinical studies show that plasma-derived exosomal metabolites like succinate and lactate correlate with response to neoadjuvant chemotherapy [126]. However, the degree of overlap or correlation between sEV-derived metabolites and those present in plasma or conditioned media from cell lines is not yet fully understood. Currently, there are limited comparative studies directly addressing these relationships in BC, which constitutes a significant knowledge gap. Future investigations employing integrated analyses of plasma, cell lines, and sEVs may provide a more comprehensive view of metabolic alterations and aid in the identification of robust and disease-specific metabolic biomarkers.

In addition, significant progress has been made in characterizing the metabolic reprogramming associated with different molecular BC subtypes, several important limitations must be acknowledged. First, tumor heterogeneity, both inter- and intra-subtype, can obscure clear metabolic signatures, particularly in clinical samples with mixed cellular compositions. Second, many studies rely on in vitro models or xenografts, which may not fully capture the metabolic dynamics of tumors in the human microenvironment. Third, variability in analytical platforms, sample handling, normalization methods, and statistical approaches across metabolomic studies can limit direct comparability and reproducibility. In addition, some reports lack robust validation cohorts or functional follow-up experiments, making it difficult to distinguish causative metabolic drivers from correlative changes. Finally, the influence of host metabolism, treatment history, and comorbidities can further complicate interpretation, especially in clinical metabolomic data. Future studies with larger, well-characterized patient cohorts, integrated multi-omics approaches, and longitudinal designs will be critical to overcome these challenges and refine our understanding of subtype-specific metabolic phenotypes.

## 7. Conclusions

Metabolomics has emerged as a modernizing field in BC research, generating original data on tumor metabolism, early diagnosis, prognosis, and personalized treatment alternatives. The current review focused on the most noteworthy advances in metabolomics, comprising the discovery of metabolic biomarkers, clarifying the involvement of metabolic pathways in tumor progression, and integrating metabolomics with other omics approaches towards precision medicine. Metabolomic profiling techniques such as MS and NMR contribute to the discovery of metabolic alterations specific to different BC subtypes. The discovery of metabolite-based biomarkers has also demonstrated excellent potential in improving early detection, predicting treatment response, and identifying therapeutic targets. Additionally, metabolic processes such as glycolysis, lipid metabolism, and glutaminolysis have also been implicated in BC development and therapy resistance, paving the path for innovative therapeutic approaches. In addition, by integrating metabolomics with AI and machine learning, predictive models can be developed that optimize biomarker discovery and personalize treatment approaches, enhancing the potential for real-time disease progression and treatment response monitoring with minimally invasive interventions.

These prospects must be confronted by confronting such crucial metabolomics challenges as method standardization, validation of large-scale clinical trial-based biomarkers, and joining hands with other omics technologies (e.g., proteomics, genomics). Complex computation structures and computations performed through artificial intelligence will also remain important promoters for driving further interpretation of the metabolomics data to a greater level, hence providing greater diagnostic return as well as stratification of the patients.

To bridge the gap between real-world clinical practice and research, increased interaction among clinicians, researchers, and regulatory agencies is necessary. Large-scale validation studies, regulation support, and incorporation of real-world evidence will be key to bringing metabolomics-based approaches into mainstream BC management. Overcoming these barriers will enable metabolomics to become a cornerstone of precision oncology, enabling more precision-driven, individualized, and streamlined therapeutic strategies in BC patients.

## Figures and Tables

**Table 1:** Some biomarkers for BC diagnosis.

Biological Fluids	Stage/Subtype	Analytical Tools	Main Conclusions	Ref.
Alveolar breath (*n* = 149)	13 Ductal carcinoma in situ (DCIS) 31 lymph node metastasis-negative 27 lymph node metastasis-positive 78 controls	GC-MS	(S)-1,2-propanediol, cyclopentanone, ethylene carbonate, 3-methoxy-1,2-propanediol, 3-methylpyridine, phenol, and tetramethylsilane can be used for early BC diagnosis VOC set showed 80.8% sensitivity and 100% specificity in identifying BC	[10]
Plasma (*n* = 270)	2 IA 54 IIA 48 IIB 15 IIIA 6 IIIB 4 IV 5 n.d. 136 controls	LC−HRMS	Two candidate biomarkers were identified with strong discriminatory power (VIP > 1 and AUC = 0.958), suggesting their potential for early diagnosis and molecular stratification of BC in future studies.	[11]
Plasma (*n* = 125)	31 I 33 II 11 III 20 controls 30 benign patients	UHPLC-MS/MS	Identified 47 plasma metabolites, including sphingomyelins, glutamate, and cysteine, as potential diagnostic biomarkers for BC. These metabolites likewise demonstrated a reasonably high predictive power in the testing cohort between benign vs. control (AUC = 0.879) and BC vs. control (AUC = 0.794).	[12]
Plasma (*n* = 44)	1 T0 1 T1a 1 T1b 5 T1c 4 T2 1 Tis 7N0 5 N1a 1 Nx 18 benign patients	LC-MS/MS	Ether-linked phosphatidylcholine showed a significant difference between invasive ductal carcinoma and benign tumors. Dysregulated hydrophilic metabolites included glutamate, glycochenodeoxycholate, and dimethyluric acid Machine learning models accurately distinguished between cancerous and benign cases using these metabolic markers.	[13]
Urine (*n*= 168)	80 biopsy-confirmed BC patients 88 controls	GC-HRMS	Variable selection (VIP > 1.5, *p* < 0.05 and FDR < 0.05) identified eight potential VOC biomarkers. Of these, three VOCs showed upregulation, and the remaining five VOCs showed downregulation in BC patients. VOC set showed 76.3% sensitivity and 85.4% specificity in identifying BC.	[14]
Urine (*n* = 60) Tissue (*n*= 60)	5 IA 10 IIA 1 IIIA 7 IIB 5 IIIB 2 IIIC 30 controls	GC-MS NMR	Acetone, 3-hexanone, 4-heptanone, 2-methyl-5-(methylthio)-furan and acetate can be potential BC biomarkers using this dual-platform approach.	[15]
Tissue (*n* = 30)	5 IA 10IIA 1 IIIA 7 IIB 5 IIIB 2 IIIC 30 controls	GC-MS	Limonene, decanoic acid, acetic acid, and furfural were identified as potential BC biomarkers with strong discriminatory power (VIP > 1 and AUC = 0.966).	[16]
Saliva (*n* = 162)	23 Tis 44 I 46 II 5 III 2 IV 42 Controls	CE LC- MS	Of the 260 quantified metabolites, polyamines were significantly elevated in the saliva of patients with breast cancer. Spermine showed the highest area under the receiver operating characteristic curves. In addition to spermine, polyamines and their acetylated forms were elevated in IC only.	[17]
Saliva (*n* = 106)	66 confirmed BC patients 40 controls	GC-MS	3-methyl-pentanoic acid, 4-methyl-pentanoic acid, phenol and p-tert-butyl-phenol (Portuguese samples) and acetic, propanoic, benzoic acids, 1,2-decanediol, 2-decanone, and decanal (Indian samples), statistically relevant for the discrimination of BC patients in the populations analyzed.	[18]

**Table 2:** Studies exploring metabolic reprogramming mechanisms in BC.

Subjects	Main Conclusions	Ref.
Cell lines (MDA-MB-231, SKBR3, MDA-MB-468)	Identified B7-H3 as a key promoter of metabolic reprogramming in cancer cells, suggesting potential for targeting B7-H3 in cancer therapy beyond immune modulation.	[46]
Cell lines (MCF-7-CSC) Mice (*n* = 7)	Targeting aberrant lipid metabolism, especially SCD1 activity, is a viable strategy to impair BC stem cell function, and omega-3 fatty acids offer a potent, non-toxic approach for this purpose.	[47]
Cell line (MET-1 cells) Mice (*n* = 60)	The study demonstrates that jointly reducing systemic glucose through diet and pharmacology can effectively inhibit tumor progression and enhance survival.	[48]
Cell lines (MDA-MB-231, MDA-MB-468) Mice (*n* = -)	Mimicking fasting conditions reduced cell proliferation, disrupted cell cycle progression, and decreased migration and invasion of TNBC cells. Intermittent fasting significantly reduced macrophage accumulation, pro-inflammatory signaling, and expression of key markers (cyclin B1, vimentin), reflecting a less aggressive tumor environment.	[49]
Tissue (*n* = -)	Metabolic differences between normal and tumor tissues were not primarily driven by tissue heterogeneity, suggesting intrinsic tumor-specific metabolic reprogramming. C3-TAg tumors exhibited a unique 10-metabolite signature with prognostic value in human BC. Gene expression analysis identified candidate genes potentially driving metabolic reprogramming in tumors.	[50]
Persons (*n* = 20)	Significant improvements were observed in fasting plasma glucose, insulin levels, and insulin resistance after three months, with these effects persisting at six months. The well-formulated ketogenic diet was successfully transitioned from a supervised to a self-administered model in Phase II, indicating potential for long-term adherence with appropriate support.	[51]
Persons (*n* = 45)	Short-term (30-day) eicosapentaenoic acid and docosahexaenoic acid supplementation led to beneficial changes in plasma fatty acid composition, immune preservation, and reduced inflammatory progression, supporting its role as a nutritional and immunological support in early-stage BC patients.	[52]
Persons (*n* = 625)	Meta-analysis revealed that intermittent fasting significantly reduced body weight, blood glucose levels, and insulin concentrations. No significant increase in chemotherapy-related adverse effects, indicating that intermittent fasting may be safe during cancer treatment, though evidence is inconclusive.	[53]
Persons (*n* = 32)	A whole-food, plant-based diet specifically impacts isoflavone and polyunsaturated fatty acid (omega-3 and 6) intake in women with advanced BC, which are associated with potential BC benefits.	[54]

**Table 3:** Summary of drug resistance to chemotherapeutic agents.

Biological Fluids	Analytical Tools	Main Conclusions	Ref.
Cell lines (MCF-7)	NMR	Metabolic and genetic markers may serve as potential targets or predictors for overcoming tamoxifen resistance in BC therapy.	[73]
Serum (*n* = 120)	GC-MS LC-MS	Thirty-nine dysregulated pathways were uncovered in 9 patients, providing deep insights into HER2+ BrCa biology and treatment resistance mechanisms. Paves the way for developing novel treatment targets for patients resistant to the TCbHP (taxane, carboplatin, trastuzumab, and pertuzumab) regimen.	[74]
Tissue (*n* = 76)	qRT-PCR	Silencing circHIPK3 can overcome paclitaxel resistance in BC by regulating the miR-1286/HK2 pathway, suggesting a potential therapeutic target.	[75]

**Table 4:** Overview of personalized therapy in BC.

Biological Samples	Molecular Subtype	Analytical Tools	Main Conclusions	Ref.
Cell lines (BT-474)	Triple-positive BC cell model	LC-MS/MS	Tamoxifen and trastuzumab (separately or in combination) exert potent anti-growth effects by modulating key pathways associated with cell proliferation, apoptosis, metabolism, and chemoresistance in triple-positive BC cells. These insights may guide the development of more personalized and less aggressive therapeutic strategies.	[124]
Cell lines (BT-20, BT-549, Hs578T, HCC38, HCC1806, HCC70, MDA-MB-231, MDA-MB-436, HMC-1–8, HCC1395, HCC1187, Hs739.T, MDA-MB-468, HCC1954, MCF-7, Hs343.T, HCC1428, DU4475, AU-565, T47D, Sk-Br-3, MDA-MB-175-VII)	Triple-negative BC cell model	LC-MS/MS	CB-839 exhibited significant antitumor activity in two xenograft models: a patient-derived TNBC model and a HER2(+) basal-like model (JIMT-1), both as a monotherapy and in combination with paclitaxel. Strong rationale for clinical development of CB-839 as a targeted therapy for TNBC and other glutamine-dependent cancers.	[104]
Plasma, tissue (*n* = 999)	Plasma: 200 BC 100 Controls Training cohort: 283 BC and 140 controls test cohort: 150 BC and 126 controls	scRNA-seq LC-MS/MS	Distinguished metabolic and immune features between TNBC and non-TNBC patients. Nucleotide metabolism correlated with regulatory T-cell activation in the tumor microenvironment via the A2AR-Treg pathway. Inosine and uridine predict response to neoadjuvant chemotherapy in TNBC patients.	[36]
Plasma (*n* = 165)	2 IA 54 IIA 15 IIIA 50 IIB 6 IIIB 4 IC 34 controls	LC-HRMS	Identified specific metabolite panels for each BC subtype: 5 metabolites for LA, 7 for LB, 5 for HER2+ and 3 for TN. The data obtained showed the clinical utility of metabolomics for individualized diagnosis and therapy planning, contributing to personalized medicine in BC.	[125]
Plasma (*n* = 16)	1 IA 3 IIA 2 IIIA 1 IB 4 IIB 3 IIIB 2 IIIC	LC-MS	Only 30% of patients achieved pathologic complete response (pCR); the rest had residual disease (RD). Plasma exosomal metabolomics could serve as a non-invasive biomarker to predict neoadjuvant chemotherapy response.	[126]
Plasma (*n* = 92)	48 LB 23 HER2+ 21 TN	LC-HRMS	Metabolomics demonstrated potential for early detection of chemoresistance. Findings contribute to advancing personalized treatment and follow-up strategies in BC care.	[127]
Urine, serum (*n* = 22)	11 BC 11 controls	NMR	Identified 9 significantly altered serum metabolites (e.g., choline, glucose, histidine) and 3 significantly altered urine metabolites (phenylacetylglycine, guanidoacetate, citrate) in BC patients. NMR-based metabolomics shows promise as a diagnostic or monitoring tool for BC.	[128]
Serum (*n* = 52)	1 I 8 II 18 III 25 IV	NMR	Three significantly altered metabolic pathways were identified that are associated with chemotherapy response. Potential of metabolic phenotyping can be used as a tool to guide personalized treatment strategies for TNBC, especially in determining suitability for neoadjuvant chemotherapy.	[129]
Serum (*n* = 35)	18 IIIA 1 IIIB 16 IIIC	LC-MS	9 key metabolites associated with chemotherapy response were identified (e.g., oleic acid amide, ethyl docosahexaenoate). Serum metabolomics can be applied as a non-invasive tool to predict neoadjuvant chemotherapy outcomes in BC.	[130]
Serum (*n* = 322)	161 BC 161 controls	NMR ICP-EOS	24 metabolites and 4 metal ions significantly differentiated BC patients from controls. Four metabolites linked to BC progression. Significant differences across age/menopausal subgroups.	[26]
Serum (*n* = 50)	22 IIB 13 IIIA 11 IIIB 4 IIIC	LC-MS/MS	Metabolic changes associated with response to neoadjuvant chemotherapy were identified, providing potential predictive biomarkers.	[131]
Serum (*n* = 50)	7 IIA 9 IIB 10 IIIA 9 IIIB 15 controls	LC-MS/MS	Metabolites allowed the differentiation between invasive ductal carcinoma patients and healthy controls, which aids in diagnosis and potentially in assessing therapy response.	[132]

## Data Availability

No new data were created or analyzed in this study.

## Abbreviations

The following abbreviations are used in this manuscript:

BC	Breast cancer
MS	Mass spectrometry
NMR	Nuclear magnetic resonance
IARC	International Agency for Research on Cancer
DCIS	Ductal carcinoma in situ
CE	Capillary electrophoresis
LC	Lung cancer
TNBC	Triple-negative breast cancer
GC-MS	Gas chromatography–mass spectrometry
LC−HRMS	Liquid chromatography coupled with high-resolution mass spectrometry
UHPLC-MS/MS	Ultra-high-performance liquid chromatography coupled with mass spectrometry
LC−MS/MS	Liquid chromatography coupled with mass spectrometry
GC-HRMS	Gas chromatography coupled with high-resolution mass spectrometry
CTL	Control
MRI	Magnetic resonance imaging
FASN	Fatty acid synthase
TCA	Tricarboxylic acid
HKII	Hexokinase II
M2 PKM2	Pyruvate kinase
LDHA	Lactate dehydrogenase A
FAO	Fatty acid oxidation
RT-qPCR	Real-time quantitative reverse transcription polymerase chain reaction
ROS	Reactive oxygen species
HIF-1α	Hypoxia-inducible factor 1-alpha
KD	Ketogenic diet
scRNA-seq	Single-cell RNA sequencing
ICP-EOS	Inductively coupled plasma optical emission spectroscopy
AUROC	Receiver operating characteristic curve
CTBP2	C-terminal binding protein 2
HR-MAS	High-resolution magic angle spinning

## References

[1] Bray F., Laversanne M., Sung H., Ferlay J., Siegel R.L., Soerjomataram I., Jemal A. (2024). Global Cancer Statistics 2022: GLOBOCAN Estimates of Incidence and Mortality Worldwide for 36 Cancers in 185 Countries. CA Cancer J. Clin..

[2] Arnold M., Morgan E., Rumgay H., Mafra A., Singh D., Laversanne M., Vignat J., Gralow J.R., Cardoso F., Siesling S. (2022). Current and Future Burden of Breast Cancer: Global Statistics for 2020 and 2040. Breast.

[3] Orrantia-Borunda E., Anchondo-Nuñez P., Acuña-Aguilar L.E., Gómez-Valles F.O., Ramírez-Valdespino C.A. (2022). Subtypes of Breast Cancer. Breast Cancer.

[4] Xiong X., Zheng L.W., Ding Y., Chen Y.F., Cai Y.W., Wang L.P., Huang L., Liu C.C., Shao Z.M., Yu K. (2025). Da Breast Cancer: Pathogenesis and Treatments. Signal Transduct. Target. Ther..

[5] Danzi F., Pacchiana R., Mafficini A., Scupoli M.T., Scarpa A., Donadelli M., Fiore A. (2023). To Metabolomics and beyond: A Technological Portfolio to Investigate Cancer Metabolism. Signal Transduct. Target. Ther..

[6] Kohli M., Poulogiannis G. (2025). Harnessing the Power of Metabolomics for Precision Oncology: Current Advances and Future Directions. Cells.

[7] Zhang T.N., Wen R., Yang Y.H., Yang N., Liu C.F. (2023). Integration of Transcriptomic, Proteomic, and Metabolomic Data to Identify LncRNA RPvt1 Associations in Lipopolysaccharide-Treated H9C2 Cardiomyocytes. Front. Genet..

[8] Wishart D.S. (2019). Metabolomics for Investigating Physiological and Pathophysiological Processes. Physiol. Rev..

[9] Qiu S., Cai Y., Yao H., Lin C., Xie Y., Tang S., Zhang A. (2023). Small Molecule Metabolites: Discovery of Biomarkers and Therapeutic Targets. Signal Transduct. Target. Ther..

[10] Zhang Y., Guo L., Qiu Z., Lv Y., Chen G., Li E. (2020). Early Diagnosis of Breast Cancer from Exhaled Breath by Gas Chromatography-Mass Spectrometry (GC/MS) Analysis: A Prospective Cohort Study. J. Clin. Lab. Anal..

[11] González Olmedo C., Díaz Beltrán L., Madrid García V., Palacios Ferrer J.L., Cano Jiménez A., Urbano Cubero R., Pérez del Palacio J., Díaz C., Vicente F., Sánchez Rovira P. (2024). Assessment of Untargeted Metabolomics by Hydrophilic Interaction Liquid Chromatography−Mass Spectrometry to Define Breast Cancer Liquid Biopsy-Based Biomarkers in Plasma Samples. Int. J. Mol. Sci..

[12] An R., Yu H., Wang Y., Lu J., Gao Y., Xie X., Zhang J. (2022). Integrative Analysis of Plasma Metabolomics and Proteomics Reveals the Metabolic Landscape of Breast Cancer. Cancer Metab..

[13] Anh N.K., Lee A., Phat N.K., Yen N.T.H., Thu N.Q., Tien N.T.N., Kim H.S., Kim T.H., Kim D.H., Kim H.Y. (2024). Combining Metabolomics and Machine Learning to Discover Biomarkers for Early-Stage Breast Cancer Diagnosis. PLoS ONE.

[14] Li X., Wen X., Luo Z., Tian Y., Qian C., Zhang J., Ling R., Duan Y. (2023). Development of a Headspace-Solid Phase Microextraction Gas Chromatography-High Resolution Mass Spectrometry Method for Analyzing Volatile Organic Compounds in Urine: Application in Breast Cancer Biomarker Discovery. Clin. Chim. Acta.

[15] Silva C.L., Perestrelo R., Capelinha F., Tomás H., Câmara J.S. (2021). An Integrative Approach Based on GC–QMS and NMR Metabolomics Data as a Comprehensive Strategy to Search Potential Breast Cancer Biomarkers. Metabolomics.

[16] Silva C., Perestrelo R., Silva P., Capelinha F., Tomás H., Câmara J.S. (2019). Volatomic Pattern of Breast Cancer and Cancer-Free Tissues as a Powerful Strategy to Identify Potential Biomarkers. Analyst.

[17] Murata T., Yanagisawa T., Kurihara T., Kaneko M., Ota S., Enomoto A., Tomita M., Sugimoto M., Sunamura M., Hayashida T. (2019). Salivary Metabolomics with Alternative Decision Tree-Based Machine Learning Methods for Breast Cancer Discrimination. Breast Cancer Res. Treat..

[18] Cavaco C., Pereira J.A.M., Taunk K., Taware R., Rapole S., Nagarajaram H., Câmara J.S. (2018). Screening of Salivary Volatiles for Putative Breast Cancer Discrimination: An Exploratory Study Involving Geographically Distant Populations. Anal. Bioanal. Chem..

[19] Birhanu A.G. (2023). Mass Spectrometry-Based Proteomics as an Emerging Tool in Clinical Laboratories. Clin. Proteom..

[20] Almalki A.H. (2023). Recent Analytical Advances for Decoding Metabolic Reprogramming in Lung Cancer. Metabolites.

[21] Roca M., Alcoriza M.I., Garcia-Cañaveras J.C., Lahoz A. (2021). Reviewing the Metabolome Coverage Provided by LC-MS: Focus on Sample Preparation and Chromatography-A Tutorial. Anal. Chim. Acta.

[22] Emwas A.H., Roy R., McKay R.T., Tenori L., Saccenti E., Nagana Gowda G.A., Raftery D., Alahmari F., Jaremko L., Jaremko M. (2019). NMR Spectroscopy for Metabolomics Research. Metabolites.

[23] Peng Y., Zhang Z., He L., Li C., Liu M. (2024). NMR Spectroscopy for Metabolomics in the Living System: Recent Progress and Future Challenges. Anal. Bioanal. Chem..

[24] Silva C.L., Olival A., Perestrelo R., Silva P., Tomás H., Câmara J.S. (2019). Untargeted Urinary1H NMR-Based Metabolomic Pattern as a Potential Platform in Breast Cancer Detection. Metabolites.

[25] Jacob M., Lopata A.L., Dasouki M., Abdel Rahman A.M. (2019). Metabolomics toward Personalized Medicine. Mass. Spectrom. Rev..

[26] Wojtowicz W., Tarkowski R., Olczak A., Szymczycha-Madeja A., Pohl P., Maciejczyk A., Trembecki, Matkowski R., Młynarz P. (2024). Serum Metabolite and Metal Ions Profiles for Breast Cancer Screening. Sci. Rep..

[27] Nagandla H., Thomas C. (2024). Estrogen Signals through ERβ in Breast Cancer; What We Have Learned since the Discovery of the Receptor. Receptors.

[28] Sirocchi C., Biancucci F., Donati M., Bogliolo A., Magnani M., Menotta M., Montagna S. (2024). Exploring Machine Learning for Untargeted Metabolomics Using Molecular Fingerprints. Comput. Methods Programs Biomed..

[29] Galal A., Talal M., Moustafa A. (2022). Applications of Machine Learning in Metabolomics: Disease Modeling and Classification. Front. Genet..

[30] Yao N., Li W., Xu G., Duan N., Yu G., Qu J. (2023). Choline Metabolism and Its Implications in Cancer. Front. Oncol..

[31] Hirschhaeuser F., Sattler U.G.A., Mueller-Klieser W. (2011). Lactate: A Metabolic Key Player in Cancer. Cancer Res..

[32] Lv Y., Yang X., Song Y., Yang D., Zheng K., Zhou S., Xie H., Guo R., Tang S. (2024). The Correlation Between Essential Amino Acid Tryptophan, Lysine, Phenylalanine and Chemotherapy of Breast Cancer. Technol. Cancer Res. Treat..

[33] Gadwal A., Panigrahi P., Khokhar M., Sharma V., Setia P., Vishnoi J.R., Elhence P., Purohit P. (2024). A Critical Appraisal of the Role of Metabolomics in Breast Cancer Research and Diagnostics. Clin. Chim. Acta.

[34] Koopaie M., Kolahdooz S., Fatahzadeh M., Manifar S. (2022). Salivary Biomarkers in Breast Cancer Diagnosis: A Systematic Review and Diagnostic Meta-analysis. Cancer Med..

[35] Dougan M.M., Li Y., Chu L.W., Haile R.W., Whittemore A.S., Han S.S., Moore S.C., Sampson J.N., Andrulis I.L., John E.M. (2018). Metabolomic Profiles in Breast Cancer: A Pilot Case-Control Study in the Breast Cancer Family Registry. BMC Cancer.

[36] Song H., Tang X., Liu M., Wang G., Yuan Y., Pang R., Wang C., Zhou J., Yang Y., Zhang M. (2024). Multi-Omic Analysis Identifies Metabolic Biomarkers for the Early Detection of Breast Cancer and Therapeutic Response Prediction. iScience.

[37] Schmidt D.R., Patel R., Kirsch D.G., Lewis C.A., Vander Heiden M.G., Locasale J.W. (2021). Metabolomics in Cancer Research and Emerging Applications in Clinical Oncology. CA Cancer J. Clin..

[38] Kaur R., Gupta S., Kulshrestha S., Khandelwal V., Pandey S., Kumar A., Sharma G., Kumar U., Parashar D., Das K. (2024). Metabolomics-Driven Biomarker Discovery for Breast Cancer Prognosis and Diagnosis. Cells.

[39] Yu D., Zhong Q., Wang Y., Yin C., Bai M., Zhu J., Chen J., Li H., Hong W. (2025). Lactylation: The Metabolic Accomplice Shaping Cancer’s Response to Radiotherapy and Immunotherapy. Ageing Res. Rev..

[40] Chen J., Huang Z., Chen Y., Tian H., Chai P., Shen Y., Yao Y., Xu S., Ge S., Jia R. (2025). Lactate and Lactylation in Cancer. Signal Transduct. Target. Ther..

[41] Saadatmand S., Geuzinge H.A., Rutgers E.J.T., Mann R.M., de Roy van Zuidewijn D.B.W., Zonderland H.M., Tollenaar R.A.E.M., Lobbes M.B.I., Ausems M.G.E.M., van ’t Riet M. (2019). MRI versus Mammography for Breast Cancer Screening in Women with Familial Risk (FaMRIsc): A Multicentre, Randomised, Controlled Trial. Lancet Oncol..

[42] Shi J., Li J., Gao Y., Chen W., Zhao L., Li N., Tian J. (2025). The Screening Value of Mammography for Breast Cancer: An Overview of 28 Systematic Reviews with Evidence Mapping. J. Cancer Res. Clin. Oncol..

[43] Suri G.S., Kaur G., Carbone G.M., Shinde D. (2023). Metabolomics in Oncology. Cancer Rep..

[44] Faubert B., Solmonson A., DeBerardinis R.J. (2020). Metabolic Reprogramming and Cancer Progression. Science.

[45] de Visser K.E., Joyce J.A. (2023). The Evolving Tumor Microenvironment: From Cancer Initiation to Metastatic Outgrowth. Cancer Cell.

[46] Lim S., Liu H., Da Silva L.M., Arora R., Liu Z., Phillips J.B., Schmitt D.C., Vu T., Mcclellan S., Lin Y. (2016). Immunoregulatory Protein B7-H3 Reprograms Glucose Metabolism in Cancer Cells by ROS-Mediated Stabilization of HIF1α. Cancer Res..

[47] Luo H., Chen C.-Y., Li X., Zhang X., Su C.-W., Liu Y., Cao T., Hao L., Wang M., Kang J.X. (2021). Increased Lipogenesis Is Critical for self-renewal and Growth of Breast Cancer Stem Cells: Impact of Omega-3 Fatty Acids. Stem Cells.

[48] Schmidt K., Thatcher A., Grobe A., Broussard P., Hicks L., Gu H., Ellies L.G., Sears D.D., Kalachev L., Kroll E. (2024). The Combined Treatment with Ketogenic Diet and Metformin Slows Tumor Growth in Two Mouse Models of Triple Negative Breast Cancer. Transl. Med. Commun..

[49] Son D.-S., Done K.A., Son J., Izban M.G., Virgous C., Lee E.-S., Adunyah S.E. (2024). Intermittent Fasting Attenuates Obesity-Induced Triple-Negative Breast Cancer Progression by Disrupting Cell Cycle, Epithelial–Mesenchymal Transition, Immune Contexture, and Proinflammatory Signature. Nutrients.

[50] Dai C., Arceo J., Arnold J., Sreekumar A., Dovichi N.J., Li J., Littlepage L.E. (2018). Metabolomics of Oncogene-Specific Metabolic Reprogramming during Breast Cancer. Cancer Metab..

[51] Buga A., Harper D.G., Sapper T.N., Hyde P.N., Fell B., Dickerson R., Stoner J.T., Kackley M.L., Crabtree C.D., Decker D.D. (2024). Feasibility and Metabolic Outcomes of a Well-Formulated Ketogenic Diet as an Adjuvant Therapeutic Intervention for Women with Stage IV Metastatic Breast Cancer: The Keto-CARE Trial. PLoS ONE.

[52] Paixão E.M.d.S., Oliveira A.C.d.M., Pizato N., Muniz-Junqueira M.I., Magalhães K.G., Nakano E.Y., Ito M.K. (2017). The Effects of EPA and DHA Enriched Fish Oil on Nutritional and Immunological Markers of Treatment Naïve Breast Cancer Patients: A Randomized Double-Blind Controlled Trial. Nutr. J..

[53] Liu X., Meng Q., Fan W., Ning L., Ge L. (2025). The Effects of Intermittent Fasting on Anthropometric Indices, Glycemic Profile, Chemotherapy-Related Toxicity, and Subjective Perception in Gynecological and Breast Cancer Patients: A Systematic Review and Meta-Analysis. BMC Cancer.

[54] Lee J., Campbell E.K., Culakova E., Blanchard L.M., Wixom N., Peppone L.J., Campbell T.M. (2024). Changes in the Consumption of Isoflavones, Omega-6, and Omega-3 Fatty Acids in Women with Metastatic Breast Cancer Adopting a Whole-Food, Plant-Based Diet: Post-Hoc Analysis of Nutrient Intake Data from an 8-Week Randomized Controlled Trial. Front. Nutr..

[55] Zhao T., Mu X., You Q. (2017). Succinate: An Initiator in Tumorigenesis and Progression. Oncotarget.

[56] Clay R., Li K., Jin L. (2025). Metabolic Signaling in the Tumor Microenvironment. Cancers.

[57] Liu S., Li Y., Yuan M., Song Q., Liu M. (2023). Correlation between the Warburg Effect and Progression of Triple-Negative Breast Cancer. Front. Oncol..

[58] Liao M., Yao D., Wu L., Luo C., Wang Z., Zhang J., Liu B. (2024). Targeting the Warburg Effect: A Revisited Perspective from Molecular Mechanisms to Traditional and Innovative Therapeutic Strategies in Cancer. Acta Pharm. Sin. B.

[59] Hammond N.G., Cameron R.B., Faubert B. (2024). Beyond Glucose and Warburg: Finding the Sweet Spot in Cancer Metabolism Models. Npj Metab. Health Dis..

[60] Liu B., Peng Q., Wang Y.W., Qiu J., Zhu J., Ma R. (2023). Prognostic and Clinicopathological Significance of Fatty Acid Synthase in Breast Cancer: A Systematic Review and Meta-Analysis. Front. Oncol..

[61] Vanauberg D., Schulz C., Lefebvre T. (2023). Involvement of the Pro-Oncogenic Enzyme Fatty Acid Synthase in the Hallmarks of Cancer: A Promising Target in Anti-Cancer Therapies. Oncogenesis.

[62] Chen C.I., Chan H.W., Shen C.Y., Chuang H.Y. (2024). Targeting Fatty Acid Synthase to Halt Tumor Progression and Enhance Radiosensitivity in Breast Cancer Cells. J. Med. Biol. Eng..

[63] Yoo H.C., Yu Y.C., Sung Y., Han J.M. (2020). Glutamine Reliance in Cell Metabolism. Exp. Mol. Med..

[64] Hanahan D. (2022). Hallmarks of Cancer: New Dimensions. Cancer Discov..

[65] de Heer E.C., Jalving M., Harris A.L. (2020). HIFs, Angiogenesis, and Metabolism: Elusive Enemies in Breast Cancer. J. Clin. Investig..

[66] Liberti M.V., Locasale J.W. (2016). The Warburg Effect: How Does It Benefit Cancer Cells?. Trends Biochem. Sci..

[67] Heiden M.G.V., Cantley L.C., Thompson C.B. (2009). Understanding the Warburg Effect: The Metabolic Requirements of Cell Proliferation. Science.

[68] Nong S., Han X., Xiang Y., Qian Y., Wei Y., Zhang T., Tian K., Shen K., Yang J., Ma X. (2023). Metabolic Reprogramming in Cancer: Mechanisms and Therapeutics. MedComm.

[69] Monaco M.E. (2017). Fatty Acid Metabolism in Breast Cancer Subtypes. Oncotarget.

[70] Vogel F.C.E., Chaves-Filho A.B., Schulze A. (2024). Lipids as Mediators of Cancer Progression and Metastasis. Nat. Cancer.

[71] Ma Y., Temkin S.M., Hawkridge A.M., Guo C., Wang W., Wang X.Y., Fang X. (2018). Fatty Acid Oxidation: An Emerging Facet of Metabolic Transformation in Cancer. Cancer Lett..

[72] Georgakopoulos-Soares I., Chartoumpekis D.V., Kyriazopoulou V., Zaravinos A. (2020). EMT Factors and Metabolic Pathways in Cancer. Front. Oncol..

[73] Alwahsh M., Hamadneh Y., Marchan R., Dahabiyeh L.A., Alhusban A.A., Hasan A., Alrawabdeh J., Hergenröder R., Hamadneh L. (2024). Glutathione and Xanthine Metabolic Changes in Tamoxifen Resistant Breast Cancer Cell Lines Are Mediated by Down-Regulation of GSS and XDH and Correlated to Poor Prognosis. J. Cancer.

[74] Zhang N., Huang Y., Wang G., Xiang Y., Jing Z., Zeng J., Yu F., Pan X., Zhou W., Zeng X. (2024). Metabolomics Assisted by Transcriptomics Analysis to Reveal Metabolic Characteristics and Potential Biomarkers Associated with Treatment Response of Neoadjuvant Therapy with TCbHP Regimen in HER2 + Breast Cancer. Breast Cancer Res..

[75] Ni J., Xi X., Xiao S., Xiao X. (2021). Silencing of CircHIPK3 Sensitizes Paclitaxel-Resistant Breast Cancer Cells to Chemotherapy by Regulating HK2 Through Targeting MiR-1286. Cancer Manag. Res..

[76] Gu Y., Yang R., Zhang Y., Guo M., Takehiro K., Zhan M., Yang L., Wang H. (2025). Molecular Mechanisms and Therapeutic Strategies in Overcoming Chemotherapy Resistance in Cancer. Mol. Biomed..

[77] Liu S., Zhang X., Wang W., Li X., Sun X., Zhao Y., Wang Q., Li Y., Hu F., Ren H. (2024). Metabolic Reprogramming and Therapeutic Resistance in Primary and Metastatic Breast Cancer. Mol. Cancer.

[78] Panda V.K., Mishra B., Mahapatra S., Swain B., Malhotra D., Saha S., Khanra S., Mishra P., Majhi S., Kumari K. (2025). Molecular Insights on Signaling Cascades in Breast Cancer: A Comprehensive Review. Cancers.

[79] Pavitra E., Kancharla J., Gupta V.K., Prasad K., Sung J.Y., Kim J., Tej M.B., Choi R., Lee J.H., Han Y.K. (2023). The Role of NF-ΚB in Breast Cancer Initiation, Growth, Metastasis, and Resistance to Chemotherapy. Biomed. Pharmacother..

[80] Wang Z.H., Zheng Z.Q., Jia S., Liu S.N., Xiao X.F., Chen G.Y., Liang W.Q., Lu X.F. (2022). Trastuzumab Resistance in HER2-Positive Breast Cancer: Mechanisms, Emerging Biomarkers and Targeting Agents. Front. Oncol..

[81] Currie E., Schulze A., Zechner R., Walther T.C., Farese R.V. (2013). Cellular Fatty Acid Metabolism and Cancer. Cell Metab..

[82] Flavin R., Peluso S., Nguyen P.L., Loda M. (2010). Fatty Acid Synthase as a Potential Therapeutic Target in Cancer. Future Oncol..

[83] Fisusi F.A., Akala E.O. (2019). Drug Combinations in Breast Cancer Therapy. Pharm. Nanotechnol..

[84] Li Y., Li Z. (2021). Potential Mechanism Underlying the Role of Mitochondria in Breast Cancer Drug Resistance and Its Related Treatment Prospects. Front. Oncol..

[85] Taneja N., Chauhan A., Kulshreshtha R., Singh S. (2024). HIF-1 Mediated Metabolic Reprogramming in Cancer: Mechanisms and Therapeutic Implications. Life Sci..

[86] Abdelmaksoud N.M., Abulsoud A.I., Doghish A.S., Abdelghany T.M. (2023). From Resistance to Resilience: Uncovering Chemotherapeutic Resistance Mechanisms; Insights from Established Models. Biochim. Biophys. Acta (BBA)-Rev. Cancer.

[87] Kar A., Agarwal S., Singh A., Bajaj A., Dasgupta U. (2024). Insights into Molecular Mechanisms of Chemotherapy Resistance in Cancer. Transl. Oncol..

[88] Godel M., Ortone G., Anobile D.P., Pasino M., Randazzo G., Riganti C., Kopecka J. (2021). Targeting Mitochondrial Oncometabolites: A New Approach to Overcome Drug Resistance in Cancer. Pharmaceutics.

[89] Liu T., Song S., Wang X., Hao J. (2021). Small-Molecule Inhibitors of Breast Cancer-Related Targets: Potential Therapeutic Agents for Breast Cancer. Eur. J. Med. Chem..

[90] Chhipa A.S., Patel S. (2021). Targeting Pyruvate Kinase Muscle Isoform 2 (PKM2) in Cancer: What Do We Know so Far?. Life Sci..

[91] Zhang L., Yao Y., Liu S. Targeting Fatty Acid Metabolism for Cancer Therapy. Fundam. Res..

[92] Samaan T.M.A., Samec M., Liskova A., Kubatka P., Büsselberg D. (2019). Paclitaxel’s Mechanistic and Clinical Effects on Breast Cancer. Biomolecules.

[93] Shrestha R., Johnson E., Byrne F.L. (2021). Exploring the Therapeutic Potential of Mitochondrial Uncouplers in Cancer. Mol. Metab..

[94] Zhi S., Chen C., Huang H., Zhang Z., Zeng F., Zhang S. (2024). Hypoxia-Inducible Factor in Breast Cancer: Role and Target for Breast Cancer Treatment. Front. Immunol..

[95] Mirzaei S., Ranjbar B., Tackallou S.H., Aref A.R. (2023). Hypoxia Inducible Factor-1α (HIF-1α) in Breast Cancer: The Crosstalk with Oncogenic and Onco-Suppressor Factors in Regulation of Cancer Hallmarks. Pathol. Res. Pract..

[96] Navarro C., Ortega Á., Santeliz R., Garrido B., Chacín M., Galban N., Vera I., De Sanctis J.B., Bermúdez V. (2022). Metabolic Reprogramming in Cancer Cells: Emerging Molecular Mechanisms and Novel Therapeutic Approaches. Pharmaceutics.

[97] Tufail M., Jiang C.-H., Li N. (2024). Altered Metabolism in Cancer: Insights into Energy Pathways and Therapeutic Targets. Mol. Cancer.

[98] Wang Z., Liu F., Fan N., Zhou C., Li D., Macvicar T., Dong Q., Bruns C.J., Zhao Y. (2020). Targeting Glutaminolysis: New Perspectives to Understand Cancer Development and Novel Strategies for Potential Target Therapies. Front. Oncol..

[99] Luo S., Jiang Y., Zheng A., Zhao Y., Wu X., Li M., Du F., Chen Y., Deng S., Chen M. (2022). Targeting Hypoxia-Inducible Factors for Breast Cancer Therapy: A Narrative Review. Front. Pharmacol..

[100] Stipp M.C., Acco A. (2025). C-Myc-Targeted Therapy in Breast Cancer: A Review of Fundamentals and Pharmacological Insights. Gene.

[101] Cui J., Jiang H. (2019). Prediction of Postoperative Survival of Triple-Negative Breast Cancer Based on Nomogram Model Combined with Expression of HIF-1α and c-Myc. Medicine.

[102] Wang P., Fu Y., Chen Y., Li Q., Hong Y., Liu T., Ding Z. (2020). Nomogram Personalizes and Visualizes the Overall Survival of Patients with Triple-Negative Breast Cancer Based on the Immune Genome. Biomed. Res. Int..

[103] Matés J.M., Di Paola F.J., Campos-Sandoval J.A., Mazurek S., Márquez J. (2020). Therapeutic Targeting of Glutaminolysis as an Essential Strategy to Combat Cancer. Semin. Cell Dev. Biol..

[104] Gross M.I., Demo S.D., Dennison J.B., Chen L., Chernov-Rogan T., Goyal B., Janes J.R., Laidig G.J., Lewis E.R., Li J. (2014). Antitumor Activity of the Glutaminase Inhibitor CB-839 in Triple-Negative Breast Cancer. Mol. Cancer Ther..

[105] Birnboim-Perach R., Benhar I. (2024). Using Combination Therapy to Overcome Diverse Challenges of Immune Checkpoint Inhibitors Treatment. Int. J. Biol. Sci..

[106] Yu W., Yang X., Zhang Q., Sun L., Yuan S., Xin Y. (2021). Targeting GLS1 to Cancer Therapy through Glutamine Metabolism. Clin. Transl. Oncol..

[107] Zipinotti dos Santos D., de Souza J.C., Pimenta T.M., da Silva Martins B., Junior R.S.R., Butzene S.M.S., Tessarolo N.G., Cilas P.M.L., Silva I.V., Rangel L.B.A. (2023). The Impact of Lipid Metabolism on Breast Cancer: A Review about Its Role in Tumorigenesis and Immune Escape. Cell Commun. Signal..

[108] Huang X., Liu B., Shen S. (2025). Lipid Metabolism in Breast Cancer: From Basic Research to Clinical Application. Cancers.

[109] Li D., Jin P., Cai Y., Wu S., Guo X., Zhang Z., Liu K., Li P., Hu Y., Zhou Y. (2025). Clinical Significance of Lipid Pathway-Targeted Therapy in Breast Cancer. Front. Pharmacol..

[110] Mukha D., Dessain J., O’Connor S., Pniewski K., Bertolazzi F., Patel J., Mullins M., Schug Z.T. (2024). Identification of Fasnall as a Therapeutically Effective Complex I Inhibitor. bioRxiv.

[111] Serhan H.A., Bao L., Cheng X., Qin Z., Liu C.-J., Heth J.A., Udager A.M., Soellner M.B., Merajver S.D., Morikawa A. (2024). Targeting Fatty Acid Synthase in Preclinical Models of TNBC Brain Metastases Synergizes with SN-38 and Impairs Invasion. NPJ Breast Cancer.

[112] Klement R.J. (2019). The Influence of Ketogenic Therapy on the 5 R’s of Radiobiology. Int. J. Radiat. Biol..

[113] Weber D.D., Aminzadeh-Gohari S., Tulipan J., Catalano L., Feichtinger R.G., Kofler B. (2020). Ketogenic Diet in the Treatment of Cancer–Where Do We Stand?. Mol. Metab..

[114] Marchio V., Augimeri G., Morelli C., Vivacqua A., Giordano C., Catalano S., Sisci D., Barone I., Bonofiglio D. (2025). Omega-3 Fatty Acids: Molecular Weapons against Chemoresistance in Breast Cancer. Cell Mol. Biol. Lett..

[115] Osouli-Tabrizi S., Mehdizadeh A., Naghdi M., Sanaat Z., Vahed N., Farshbaf-Khalili A. (2023). The Effectiveness of Omega-3 Fatty Acids on Health Outcomes in Women with Breast Cancer: A Systematic Review. Food Sci. Nutr..

[116] Morshed A.K.M.H., Al Azad S., Mia M.d.A.R., Uddin M.F., Ema T.I., Yeasin R.B., Srishti S.A., Sarker P., Aurthi R.Y., Jamil F. (2023). Oncoinformatic Screening of the Gene Clusters Involved in the HER2-Positive Breast Cancer Formation along with the in Silico Pharmacodynamic Profiling of Selective Long-Chain Omega-3 Fatty Acids as the Metastatic Antagonists. Mol. Divers..

[117] Anemoulis M., Vlastos A., Kachtsidis V., Karras S.N. (2023). Intermittent Fasting in Breast Cancer: A Systematic Review and Critical Update of Available Studies. Nutrients.

[118] Omar E.M., Omran G.A., Mustafa M.F., El-Khodary N.M. (2022). Intermittent Fasting during Adjuvant Chemotherapy May Promote Differential Stress Resistance in Breast Cancer Patients. J. Egypt. Natl. Canc Inst..

[119] Carmona A., Mitri S., James T.A., Ubellacker J.M. (2024). Lipidomics and Metabolomics as Potential Biomarkers for Breast Cancer Progression. npj Metab. Health Dis..

[120] Cappelletti V., Iorio E., Miodini P., Silvestri M., Dugo M., Daidone M.G. (2017). Metabolic Footprints and Molecular Subtypes in Breast Cancer. Dis. Markers.

[121] Pang H., Hu Z. (2023). Metabolomics in Drug Research and Development: The Recent Advances in Technologies and Applications. Acta Pharm. Sin. B.

[122] Seydel C. (2021). Single-Cell Metabolomics Hits Its Stride. Nat. Methods.

[123] Castelli F.A., Rosati G., Moguet C., Fuentes C., Marrugo-Ramírez J., Lefebvre T., Volland H., Merkoçi A., Simon S., Fenaille F. (2022). Metabolomics for Personalized Medicine: The Input of Analytical Chemistry from Biomarker Discovery to Point-of-Care Tests. Anal. Bioanal. Chem..

[124] Sharaf B.M., Giddey A.D., Al-Hroub H.M., Menon V., Okendo J., El-Awady R., Mousa M., Almehdi A., Semreen M.H., Soares N.C. (2022). Mass Spectroscopy-Based Proteomics and Metabolomics Analysis of Triple-Positive Breast Cancer Cells Treated with Tamoxifen and/or Trastuzumab. Cancer Chemother. Pharmacol..

[125] Díaz-Beltrán L., González-Olmedo C., Luque-Caro N., Díaz C., Martín-Blázquez A., Fernández-Navarro M., Ortega-Granados A.L., Gálvez-Montosa F., Vicente F., Pérez del Palacio J. (2021). Human Plasma Metabolomics for Biomarker Discovery: Targeting the Molecular Subtypes in Breast Cancer. Cancers.

[126] Joshi S., Garlapati C., Bhattarai S., Su Y., Rios-Colon L., Deep G., Torres M.A., Aneja R. (2022). Exosomal Metabolic Signatures Are Associated with Differential Response to Neoadjuvant Chemotherapy in Patients with Breast Cancer. Int. J. Mol. Sci..

[127] Díaz C., González-Olmedo C., Díaz-Beltrán L., Camacho J., Mena García P., Martín-Blázquez A., Fernández-Navarro M., Ortega-Granados A.L., Gálvez-Montosa F., Marchal J.A. (2022). Predicting Dynamic Response to Neoadjuvant Chemotherapy in Breast Cancer: A Novel Metabolomics Approach. Mol. Oncol..

[128] Zhou J., Wang Y., Zhang X., Zhou J., Wang Y., Zhang X. (2017). Metabonomics Studies on Serum and Urine of Patients with Breast Cancer Using 1 H-NMR Spectroscopy. Oncotarget.

[129] He X., Gu J., Zou D., Yang H., Zhang Y., Ding Y., Teng L. (2021). NMR-Based Metabolomics Analysis Predicts Response to Neoadjuvant Chemotherapy for Triple-Negative Breast Cancer. Front. Mol. Biosci..

[130] Lin X., Xu R., Mao S., Zhang Y., Dai Y., Guo Q., Song X., Zhang Q., Li L., Chen Q. (2019). Metabolic Biomarker Signature for Predicting the Effect of Neoadjuvant Chemotherapy of Breast Cancer. Ann. Transl. Med..

[131] Fang Z., Ren G., Ke S., Xu Q., Chen Y., Shi X., Guo C., Huang J. (2025). Serum Metabolomic Profiling for Predicting Therapeutic Response and Toxicity in Breast Cancer Neoadjuvant Chemotherapy: A Retrospective Longitudinal Study. Breast Cancer Res..

[132] Eniu D.T., Chiorean A.R., Socaciu A.I., Staicu A., Rachieriu C., Goidescu I., Buiga R., Eniu D., Socaciu C., Chira R. (2022). Blood and Urine Biomarkers in Invasive Ductal Breast Cancer: Mass Spectrometry Applied to Identify Metabolic Alterations. J. Mol. Struct..

[133] Zheng Y., Xu R., Chen X., Lu Y., Zheng J., Lin Y., Lin P., Zhao X., Cui L. (2024). Metabolic Gatekeepers: Harnessing Tumor-Derived Metabolites to Optimize T Cell-Based Immunotherapy Efficacy in the Tumor Microenvironment. Cell Death Dis..

[134] Santiappillai N.T., Abuhammad S., Slater A., Kirby L., McArthur G.A., Sheppard K.E., Smith L.K. (2021). CDK4/6 Inhibition Reprograms Mitochondrial Metabolism in BRAFV600 Melanoma via a P53 Dependent Pathway. Cancers.

[135] Irajizad E., Wu R., Vykoukal J., Murage E., Spencer R., Dennison J.B., Moulder S., Ravenberg E., Lim B., Litton J. (2022). Application of Artificial Intelligence to Plasma Metabolomics Profiles to Predict Response to Neoadjuvant Chemotherapy in Triple-Negative Breast Cancer. Front. Artif. Intell..

[136] Neagu A.-N., Whitham D., Buonanno E., Jenkins A., Alexa-Stratulat T., Tamba B.I., Darie C.C. (2021). Proteomics and Its Applications in Breast Cancer. Am. J. Cancer Res..

[137] Mehmood S., Faheem M., Ismail H., Farhat S.M., Ali M., Younis S., Asghar M.N. (2022). ‘Breast Cancer Resistance Likelihood and Personalized Treatment Through Integrated Multiomics’. Front. Mol. Biosci..

[138] Gómez-Cebrián N., Domingo-Ortí I., Poveda J.L., Vicent M.J., Puchades-Carrasco L., Pineda-Lucena A. (2021). Multi-Omic Approaches to Breast Cancer Metabolic Phenotyping: Applications in Diagnosis, Prognosis, and the Development of Novel Treatments. Cancers.

[139] Rossi C., Cicalini I., Cufaro M.C., Consalvo A., Upadhyaya P., Sala G., Antonucci I., Del Boccio P., Stuppia L., De Laurenzi V. (2022). Breast Cancer in the Era of Integrating “Omics” Approaches. Oncogenesis.

[140] Auslander N., Yizhak K., Weinstock A., Budhu A., Tang W., Wang X.W., Ambs S., Ruppin E. (2016). A Joint Analysis of Transcriptomic and Metabolomic Data Uncovers Enhanced Enzyme-Metabolite Coupling in Breast Cancer. Sci. Rep..

[141] Bonanomi M., Salmistraro N., Fiscon G., Conte F., Paci P., Bravatà V., Forte G.I., Volpari T., Scorza M., Mastroianni F. (2021). Transcriptomics and Metabolomics Integration Reveals Redox-Dependent Metabolic Rewiring in Breast Cancer Cells. Cancers.

[142] Xiao Y., Ma D., Yang Y.-S., Yang F., Ding J.-H., Gong Y., Jiang L., Ge L.-P., Wu S.-Y., Yu Q. (2022). Comprehensive Metabolomics Expands Precision Medicine for Triple-Negative Breast Cancer. Cell Res..

[143] González-Domínguez R., González-Domínguez Á., Sayago A., Fernández-Recamales Á. (2020). Recommendations and Best Practices for Standardizing the Pre-Analytical Processing of Blood and Urine Samples in Metabolomics. Metabolites.

[144] Ahn J.S., Shin S., Yang S.-A., Park E.K., Kim K.H., Cho S.I., Ock C.-Y., Kim S. (2023). Artificial Intelligence in Breast Cancer Diagnosis and Personalized Medicine. J. Breast Cancer.

[145] Polónia A., Campelos S., Ribeiro A., Aymore I., Pinto D., Biskup-Fruzynska M., Veiga R.S., Canas-Marques R., Aresta G., Araújo T. (2021). Artificial Intelligence Improves the Accuracy in Histologic Classification of Breast Lesions. Am. J. Clin. Pathol..

[146] Uzun Ozsahin D., Ikechukwu Emegano D., Uzun B., Ozsahin I. (2022). The Systematic Review of Artificial Intelligence Applications in Breast Cancer Diagnosis. Diagnostics.

[147] Eisemann N., Bunk S., Mukama T., Baltus H., Elsner S.A., Gomille T., Hecht G., Heywang-Köbrunner S., Rathmann R., Siegmann-Luz K. (2025). Nationwide Real-World Implementation of AI for Cancer Detection in Population-Based Mammography Screening. Nat. Med..

[148] Letertre M.P.M., Giraudeau P., de Tullio P. (2021). Nuclear Magnetic Resonance Spectroscopy in Clinical Metabolomics and Personalized Medicine: Current Challenges and Perspectives. Front. Mol. Biosci..

[149] Borges R.M., Teixeira A.M. (2024). On the Part That NMR Should Play in Mass Spectrometry Metabolomics in Natural Products Studies. Front. Nat. Prod..

[150] Shah T., Wildes F., Penet M.F., Winnard P.T., Glunde K., Artemov D., Ackerstaff E., Gimi B., Kakkad S., Raman V. (2010). Choline Kinase Overexpression Increases Invasiveness and Drug Resistance of Human Breast Cancer Cells. NMR Biomed..

[151] Carosi F., Broseghini E., Fabbri L., Corradi G., Gili R., Forte V., Roncarati R., Filippini D.M., Ferracin M. (2024). Targeting Isocitrate Dehydrogenase (IDH) in Solid Tumors: Current Evidence and Future Perspectives. Cancers.

[152] Wang Y., An R., Yu H., Dai Y., Lou L., Quan S., Chen R., Ding Y., Zhao H., Wu X. (2024). Largescale Multicenter Study of a Serum Metabolite Biomarker Panel for the Diagnosis of Breast Cancer. iScience.

[153] Bai M., Sun C. (2022). Determination of Breast Metabolic Phenotypes and Their Associations with Immunotherapy and Drug-Targeted Therapy: Analysis of Single-Cell and Bulk Sequences. Front. Cell Dev. Biol..

[154] Yoon H., Yoon D., Yun M., Choi J.S., Park V.Y., Kim E.-K., Jeong J., Koo J.S., Yoon J.H., Moon H.J. (2016). Metabolomics of Breast Cancer Using High-Resolution Magic Angle Spinning Magnetic Resonance Spectroscopy: Correlations with 18F-FDG Positron Emission Tomography-Computed Tomography, Dynamic Contrast-Enhanced and Diffusion-Weighted Imaging MRI. PLoS ONE.

[155] Ahmad Qureshi M.D., Ramzan M.F., Amjad F., Haider N. (2024). Artificial Intelligence in Metabolomics for Disease Profiling: A Machine Learning Approach to Biomarker Discovery. Indus J. Biosci. Res..

[156] Buentzel J., Klemp H.G., Kraetzner R., Schulz M., Dihazi G.H., Streit F., Bleckmann A., Menck K., Wlochowitz D., Binder C. (2021). Metabolomic Profiling of Blood-Derived Microvesicles in Breast Cancer Patients. Int. J. Mol. Sci..

[157] D’Mello R., Hüttmann N., Minic Z., V Berezovski M. (2024). Untargeted Metabolomic Profiling of Small Extracellular Vesicles Reveals Potential New Biomarkers for Triple Negative Breast Cancer. Metabolomics.

